# Design and Fabrication of Microspheres with Hierarchical Internal Structure for Tuning Battery Performance

**DOI:** 10.1002/advs.201500078

**Published:** 2015-04-21

**Authors:** Lea V. Nowack, Teutë Bunjaku, Karsten Wegner, Sotiris E. Pratsinis, Mathieu Luisier, Vanessa Wood

**Affiliations:** ^1^ ETH ZürichDepartment of Information Technology and Electrical EngineeringGloriastrasse 358092ZürichSwitzerland; ^2^ ETH ZürichDepartment of Mechanical and Process EngineeringSonneggstrasse 38092ZürichSwitzerland

**Keywords:** lithium ion batteries, microspheres, nanoparticles, rate performance, spray drying

## Abstract

**Microspheres with controlled nano‐ and macroporosity** are fabricated by template‐assisted spray drying. Increasing the porosity of the particle up to 20% improves the rate performance of the particles as shown experimentally and by electrochemical simulations of particle lithiation.

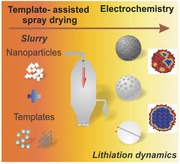

The development of higher performance lithium ion batteries (LIBs) requires not only higher capacity materials but also their rational structuring for optimal function within the LIB. This is true for both existing intercalation compounds and next generation conversion compounds; in intercalating transition metal oxides, the particle internal structure can be used to tune the trade‐off between energy density and power.^1^, ^2^ In conversion systems such as silicon and tin, such internal structure can be used to accommodate for their large volume expansion.^3^, ^4^, ^5^

Structuring of intercalation compounds has already been the subject of extensive investigation. Materials with rather slow lithium ion diffusion, such as lithium titanate (LTO), exhibit enhanced performance with nanostructuring that does not significantly impact the electron transport in the material.^6^, ^7^, ^8^, ^9^ Structuring generates a pore network that enables penetration of the liquid electrolyte into the particle, such that lithium ions are transported in the electrolyte and lithium ion have a shorter diffusion path in the solid. Assembling nanoparticles into micrometer‐sized spherical particles with a defined structure is a particularly attractive approach for fabricating active materials for LIBs both in terms of electrode manufacturing and electrochemical performance of the resulting cell.^10^, ^11^, ^12^, ^13^ Such assembled nanostructured microparticles have a higher tap density than nanopowders, which results in a higher packing density of the particles in the electrodes and therefore higher volumetric energy density.^14^, ^15^, ^16^ Furthermore, less polymeric binder and conductive agent is required to ensure an electrical path between the particles and the current collector compared to nanopowders. In addition, particle handling may be easier and safer in view of concerns associated with nanoparticles in industry.^17^ Finally, spherically shaped microparticles are advantageous over platelets or ellipsoidal‐shaped particles in terms of decreased electrode tortuosity as shown by Ebner et al.^18^

Here we investigate template assisted spray drying as a scalable fabrication method for controlling the internal structure of microspheres assembled from nanoparticles. Different techniques, including spray drying of precursor materials^10^ and hydrothermal synthesis,^19^, ^20^ have been explored to produce mesoporous spherical micrometer‐sized particles. We opt for spray drying since it is a high‐throughput and scalable technique that can be applied to essentially all material systems that start as sols^21^ or colloidal nanoparticles.^22^, ^23^, ^24^ As shown schematically in **Figure** **1**a, spray drying involves drying of viscous slurry droplets, which are atomized by a nozzle in a hot air stream. Spray drying is already widely used in industry^25^, ^26^ and has been explored for battery materials, including carbon coated LiFe_0.6_Mn_0.4_PO_4_/C 27 or different forms of LTO. 13, 28, 29

**Figure 1:**
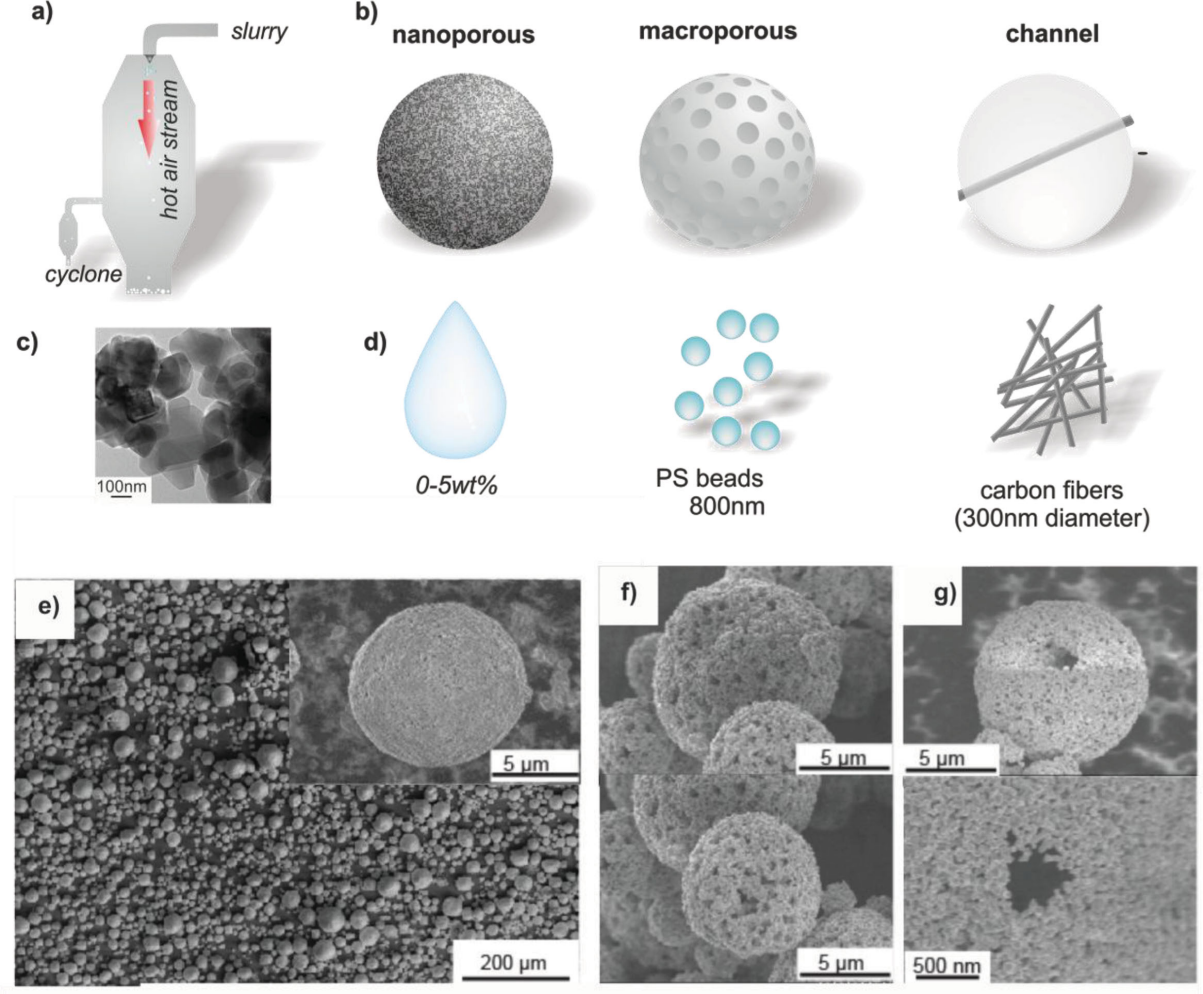
a) Schematic of the spray drying process. b) The three types of internal structure (nanoporosity, macroporosity, and channels) targeted in this study. c) TEM image of the LTO nanoparticles used for all spray drying slurries. d) Schematics of the different templates used to obtain hierarchical structures. e) Overview and close‐up SEM images of nanoporous microspheres obtained from spray drying with 3 wt% cellulose. SEM images of f) macroporous spheres obtained from spray drying with PS beads as template and g) microspheres with channel structures obtained from spray drying with carbon fiber templates.

Templates, which are removed after synthesis, are often used to obtain hollow or porous active battery materials.^4^, ^16^ Hollow structures have been fabricated for example by coprecipitation and etching^29^ or template‐assisted chemical vapor deposition for silicon.^4^ We identify three types of structural features that are of interest for electrochemical performance of LIB activity materials: 1) nanoscale porosity, 2) macroscale porosity, and 3) particle transecting channels (see Figure 1b). After demonstrating template‐assisted spray drying as a method to assemble nanoparticles into microspheres with controlled size, porosity, and hierarchical structure, we show experimentally that changes to particle structure provide us with control over the rate performance of the LIB electrode. To verify that the observed electrochemical changes are due to the controlled variation of the particle structure, we perform electrochemical simulations on single particles with representative morphologies.

To demonstrate spray drying as a technique to achieve hierarchical structure in microparticles and thereby improve electrochemical performance, we select lithium titanate (LTO) as a model system. With a theoretical capacity of 175 mAh g^−1^, zero strain insertion of lithium ions, and a higher voltage plateau of 1.55 V compared to graphite, LTO is already used as an anode material in LIBs. As detailed in the Experimental Section, our spray drying slurry consists of commercially available LTO nanoparticles 120 nm in diameter (see TEM image in Figure 1c) dispersed in deionized water. The slurry is atomized with a two‐phase nozzle into a 200 °C air coflow, resulting in micrometer‐sized particles as shown in Figure 1e. To obtain nanopores, macropores, or channels (shown schematically in Figure 1b), we add templates (cellulose, polystyrene (PS) beads, or carbon fibers (CF)) to the slurry (Figure 1d), which are removed subsequent to microparticle formation via calcination. Every batch is characterized for frequency particle size distribution (PSD) using laser diffraction, specific surface area (SSA) using nitrogen adsorption, and morphology using scanning electron microscopy (SEM). PSD and SSA results are given in **Table** **1**. To compare the electrochemical performance of the particles with different internal structure, we maintain the same PSD with a mode at ≈20 μm so that any impact of particle size variation on electrochemical performance can be minimized.

**Table 1:** Experimental conditions, SSA measurements, volume mean diameter of the PSD, and Dv 10, 50, and 90 values of the different samples. For volume distributions, the median of the distribution or the D50 value is the diameter for which half of the total particle volume is in particles smaller than this value. Similarly, 90% of the distribution is in particles with diameters below the D90 value and 10% below the D10 value

Sample	PS bead solution [mL]	Cellulose [wt%]	SSA [m^2^ g^−1^]	PSD volume mean diameter [μm]	PSD Dv10 [μm]	PSD Dv50 [μm]	PSD Dv90 [μm]	Standard deviation [μm]
Nanoparticles	–	–	9.2	–	–	–	–	–
Dense (no template)	–	0	4.5	19.3	5.4	18.1	36.2	11.9
Cellulose 3%	–	3	5.4	22.77	5.1	17.8	35.2	11.5
Cellulose 5%	–	5	6.9	19.08	5.2	17.2	35.4	11.9
PS beads	3	–	7.0	19.48	6.7	19.5	38.9	12.3
Carbon fibers	–	–	5.5	22.18	5.7	17.4	35.9	12.1

First, we tune the nanoscale porosity of the LTO microspheres by adding hydrophilic cellulose into the water‐based spray dry slurry and subsequently removing it via calcination of the resulting micrometer‐sized particles formed during the spray‐drying process. Particles before calcination are shown in Figure S1 (Supporting Information). The one hour calcination step at 750 °C does not alter the phase composition of the LTO, as shown by X‐ray diffraction (XRD) results in Figure S2 (Supporting Information). Analysis of the diffraction patterns by Rietveld refinement indicates an average crystal size of 121 nm before and 183 nm after calcination. This indicates slight sintering of the nanoparticles, which stabilizes the microspherical structure. When increasing the amount of cellulose in the slurry from 0 to 5 wt%, the PSD of the microspheres (**Figure** **2**a) remains comparable, while the nitrogen adsorption measurements (Table 1) show increasing SSA with increasing cellulose content, indicating a more extensive pore network through the microspheres.

**Figure 2:**
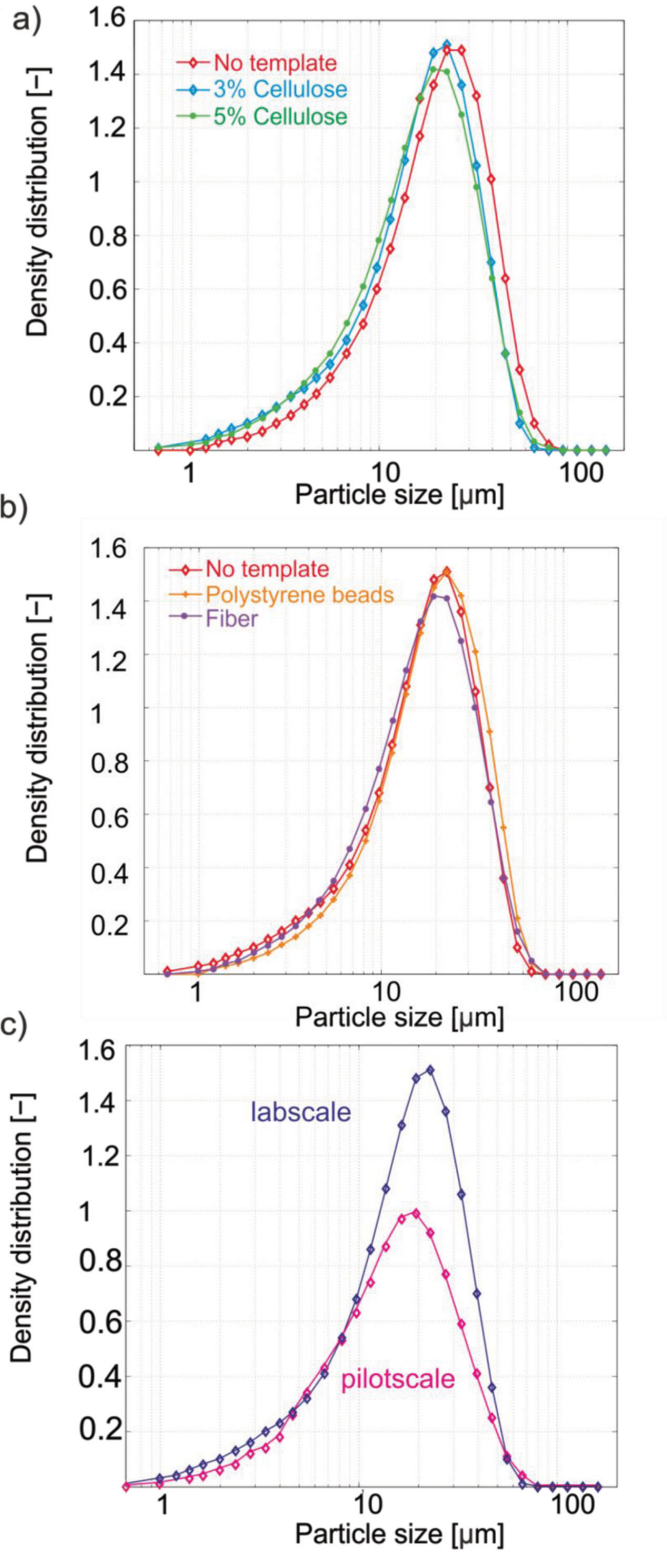
a) Volume‐based particle size frequency distributions (PSDs) of dense particles (no templates) and those fabricated with 3 and 5 wt% cellulose. b) PSDs of dense particles and those fabricated with PS and CF templates. c) Comparison of PSD of particles obtained with the laboratory and pilot‐scale spray dryers without templates.

Second, to achieve microspheres with macroscale pores or channels, we add either spherical 800 nm diameter PS beads or high aspect ratio carbon fibers as templates into the slurry as shown schematically in Figure 1. We select the size of the PS beads following Iskandar et al.,^30^, ^31^ who studied porous microparticles obtained by spray drying a slurry of silica nanoparticles and latex beads and showed that stable microparticles are achieved when the ratio of the nanoparticle size to the template diameter is close to 0.15. In our case, with LTO nanoparticles 120 nm in diameter, we choose 800 nm diameter templates, which results in a ratio of 0.125. As with cellulose, these PS templates are removed during calcination of the spray dried microspheres at 750 °C in air. As seen in the SEM images in Figure 1f, PS bead templates result in microspheres with evenly distributed holes that are comparable to the size of the removed PS beads. In the case of carbon fiber (CF) templates, we obtain particles with on average one or two well‐defined channels that traverse the particles (Figure 1g). Again, the PSDs (Figure 2b) remain comparable between the batches of microspheres containing the different templates, while SSAs are similar to nanoporous particles formed using cellulose as a template (Table 1).

To demonstrate the scalability of the template‐based spray drying approach, we also fabricate particles in a pilot‐scale spray dryer^32^, ^33^ capable of a production rate of 4 kg h^−1^. Figure 2c compares the PSDs of particles fabricated with the lab scale and pilot‐scale systems, showing that it is possible to achieve similar PSDs from the same starting slurry. Since the pilot‐scale system requires large volumes (i.e., liters) of slurry, it is not practical for carrying out the comparative study conducted here, but this trial with the pilot‐scale system highlights the ease in up‐scaling the lab‐based procedures shown here.

Using the microspheres of the same average particle size, but with different internal porosities and structures, we fabricate electrodes as detailed in the Experimental Section and assess their electrochemical performance as a function of C‐rate (where 1C corresponds to a (dis)charge rate of 1 h) averaged over four samples and ten cycles for each rate. Electrodes are fabricated to be ≈60 μm thick with relatively high porosity (no calendering) so that particle microstructure, and not electrode microstructure, dominates electrochemical performance. Figure S3 (Supporting Information) shows the long term cycle stability of the different particle types.

**Figure** **3**a shows the rate performance for microparticles fabricated with 0, 3, and 5 wt% cellulose in the slurry. At low cycling rates (0.1C), all materials exhibit a capacity close to the theoretical 175 mAh g^−1^. The improvements enabled by structuring become obvious at higher rates: at 10C, nanoporous microparticles fabricated with 5 wt% cellulose exhibit capacities of about 150 mAh g^−1^, while dense microparticles fabricated without a template (i.e., 0 wt% cellulose) exhibit only 100 mAh g^−1^. Comparing Figure 3a,b, we see that macroporous particles formed using 800 nm PS templates show similar rate performance to the nanoporous particles formed using 5% cellulose, both exhibiting capacities of about 150 mAh g^−1^ at 10C. The microparticles with single channels formed with carbon fiber templates also show higher capacities at higher C‐rates (140 mAh g^−1^ at 10C) than the dense microparticles (100 mAh g^−1^ at 10C). The trend in capacity improvement at high C‐rates correlates well with the measurements of SSA (Table 1): the larger the SSA of a microparticle, the greater the achieved capacity at elevated C‐rate.

**Figure 3:**
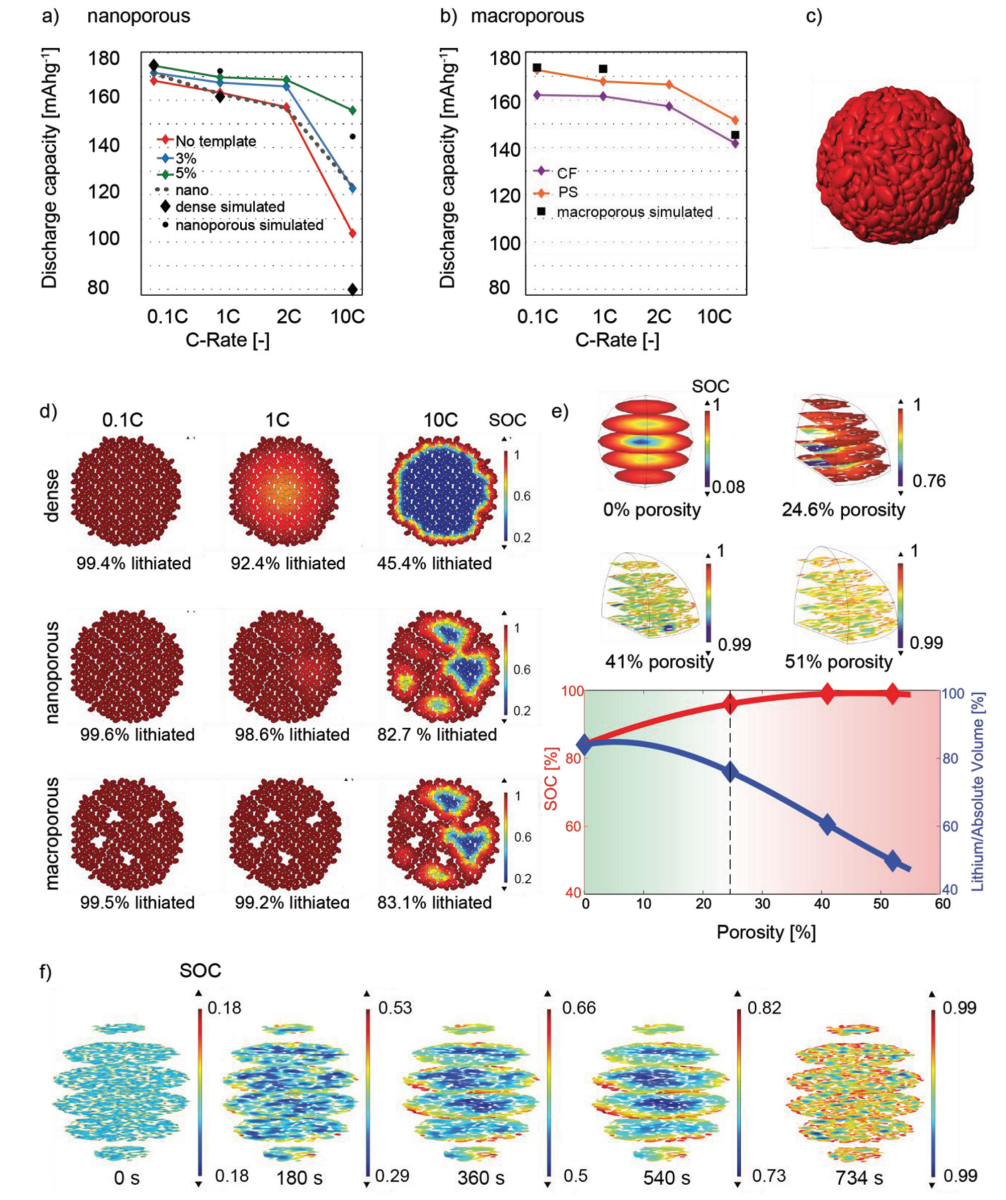
a) Experimentally measured capacities at different C‐rates for spray dried structures with no templates (red line), with 3 wt% cellulose (blue line), and 5 wt% cellulose (green line) as well as for the LTO nanoparticles (dashed gray line). b) Experimentally measured capacities at different C‐rates for spray dried structures with macroporous structures obtained with PS beads (orange line) and carbon fiber (purple line). Black points in panels (a) and (b) are from simulation of single micrometer‐sized particles with computer‐generated structures. c) Computer‐generated 3D structure of a single particle. d) Simulation of the lithium concentration in a dense (top row), nanoporous (middle row), and macroporous (bottom row) structure at the end of a galvanostatic cycles at 0.1C (left column), 1C (middle column), and 10C (right column). e) SOC distributions at the end of a galvanostatic 5C half‐cycle for a dense sphere and 1/8 of spheres with different nanoporosities. Note different SOC scale bars. Plot of final SOC versus porosity (red) and decrease in total energy density of particle (blue). e) Spatial visualization of lithiation in a structure with 41.4% porosity at different time steps during a 5C charging cycle.

The dashed gray line in Figure 3a shows the performance of the LTO nanoparticles not assembled into microspheres. The achieved capacity at 10C is 140 mAh g^−1^, similar to that of microparticles fabricated with 3 wt% cellulose or carbon fiber (CF) templates. However, we note that direct electrochemical comparison of these dispersed nanoparticles and microspheres is difficult since nanoparticles are not exposed to calcination and the electrode preparation was not optimized for nanoparticles; however, our findings are on par with literature values where electrode fabrication was optimized for nanoparticles.^34^

To corroborate that our experimental results showing improved rate capabilities for particles with higher porosity are indeed due to the internal structure within the microparticles, we perform electrochemical simulations on single particles having computer‐generated structures that mimic those created experimentally through template‐assisted spray drying. Simulations are performed in the Battery and Fuel Cell package of COMSOL Multiphysics 4.4. In analogy with the spray drying approach, computer‐generated microparticles (such as the one in Figure 3c) are first created in MATLAB from ellipsoids with different sizes and orientations. These ellipsoids are then enlarged or the number of ellipsoids is varied until the desired porosity value is reached. Given the nanoscale features, it is a challenge to experimentally quantify the porosity of our experimentally prepared microspheres, but we estimate that the addition of 3%–5% cellulose in the slurry results in microspheres with nanoporosity of 10%–20%. To prevent the unrealistic scenario of overlapping nanoparticles in the computer‐generated microsphere, a code is used to check whether the ellipsoids intersect.^35^ If any intersection occurs, the ellipsoid axes are shrunk and tested again for intersections. This is repeated until the axes do not intersect with neighboring ellipsoids or until the minimal axes size, defined beforehand by the user and corresponding to the smallest nanoparticles, is reached. Macropores or channels are also added by taking single ellipsoids away or moving them until they do not touch. Using the script described, single secondary particles with different sizes and porosities can be generated. Meshing of these structures is performed using a tetrahedral mesh in Simpleware and exported into COMSOL, where the electrochemical simulations are performed.

Lithiation and delithiation against lithium of a single particle is simulated. The simulation environment is shown in Figure S4 (Supporting Information), and parameters and boundary conditions are detailed in the Experimental Section and Supporting Information. The particle of 12 μm is placed in a rectangular volume with a homogeneous mixture of electrolyte and carbon that also infills the particle pores. As used in the experiment, one side of the rectangular volume is a lithium metal electrode with a volume of 0.16 nL. It is separated from the particle with a 30 μm thick region filled with electrolyte. In addition to 3D structures, we also perform 2D simulations, where the circle representing the active particle is generated from ellipses directly in COMSOL. By comparing discharge curves (Figure S5, Supporting Information), we find that the 2D simulations capture the key aspects of the 3D lithiation dynamics, we use mainly 2D simulations for computational efficiency and to obtain easy‐to‐interpret spatial maps of the lithiation in a single particle.

Figure 3d shows spatial maps of the state of charge (SOC) in 2D particles with internal structure representative of the experimental dense, nanoporous, and macroporous spheres. Red coloring corresponds to a SOC of 1, indicating complete lithiation, while blue corresponds to a SOC of 0.2 or lower. At 0.1C, 1C, and 10C, the final lithiations of 99.4%, 92.2%, and 45.4% for the dense particle, of 99.6%, 98.6%, and 82.7% for the nanoporous particle, and of 99.5%, 91.2%, and 83.1% for the macroporous particle correlate well with the trends in the maximum capacity seen experimentally (Figure 3a,b) where the capacities are 96.5%, 92%, and 58% for dense particles, 98.8%, 96.5%, and 88.5% for nanoporous particles, and 99%, 97%, and 89% for macroporous particles. From the spatial maps of the lithiation, we see that at high C‐rates, an interconnected pathway of pores enhances the homogeneity of the lithiation and facilitates the achievement of high capacities even at fast C‐rates. These results highlight that for materials with relatively slow solid‐state diffusion, such as LTO, pores within a microsphere can facilitate high rate cycling.^19^ Consistent with the experimental results on particles prepared with 5 wt% cellulose binder and PS bead templates showing similar capacities versus C‐rate (Figure 3a,b), the simulated capacities at different cycle rates for the nanoporous (Figure 3d, second row) and the macroporous particles (Figure 3d, third row) are comparable.

To study the tradeoff associated with increasing porosity, which enables higher capacities, but comes at the cost of lower volumetric energy densities, we focus on 3D simulations. Figure 3e,f reports the SOC achieved by galvanostatic lithiation against lithium at a 5C‐rate for 3D structures with nanoscale porosities of 0%, 27%, 41.4%, and 52%. The spatial maps and extracted capacities show, as did the 2D simulations in Figure 3d, that structures with higher porosities result in more uniform and complete lithiation. At the same time, the amount of lithium that can be stored in a volume decreases with increasing porosity (blue curve in Figure 3f). Up to 20% porosity (green shaded region), the improvement in the final achieved SOC enabled by the increasing porosity is greater than the loss in volumetric energy density. Over 20% of nanoscale porosity more energy density is lost, while the slope of obtained SOC at 5C flattens, indicating no further gains in lithiation performance with a more porous structure. Indeed, Figure 3f shows cross‐sectional slices through a particle with a porosity of 41.4%, at different times during the 5C lithiation. Even at this high rate, full lithiation is achieved and proceeds from the outside to the inside of the particle over time.

This experimental and computational work highlights that it is possible to design and fabricate microspheres of active materials in LIBs with internal structure using template‐assisted spray drying, which we show to be an industrial scalable approach. Templates that are removed subsequent to microparticle formation can be used to define a porosity within the microparticle, which facilitates lithium diffusion and increases the achieved capacity at high C‐rates. Depending on the rate capability desired for a specific application, it is possible to determine an optimal porosity where high capacities can be reached at fast (dis)charge with a minimal loss of energy density due to the increased porosity. Since template‐assisted spray drying is generalizable to a variety of starting nanoparticles or precursors, this approach could be applied to engineer a variety of battery materials ranging from intercalation compounds such as Li(Ni,Mn,Co)O_2_,^25^ Li(Ni,Co,Al)O_2_ to strongly expanding active materials such as silicon or sulfur.

## Experimental Section

*Spray Drying*: Commercial nanosized (≈120 nm) LTO particles are assembled by spray drying into microspheres of ≈20 μm in diameter (Figure 2). The spray drying slurry contains 30 wt% LTO nanoparticles in deionized water. To achieve different nanoscale porosities, we add varying amounts of hydrophilic cellulose from 0 to 5 wt%. In order to achieve macroporous structures, we add 3 mL of PS bead solution (Sigma‐Aldrich, PS beads, 10% solid 800 nm) to 10 mL of spray drying slurry, which is 10 mass% of the LTO particles (compared to 7–14 mass percent employed by Isakander et al.^30^. For the experiments containing carbon fibers, a carbon fiber web (Steinemann AG, Switzerland) is ball milled for 4 h at 1000 rpm and 200 mg of fibers are added under stirring to 10 mL of slurry (6 mass%).

For spray drying with the Büchi‐B290 system, the slurry is kept stirring and fed to the spray dryer by a tube pump. The slurry is dispersed by an air stream (40 L h^−1^) through a 0.7 mm nozzle at 200 °C. The particles are collected in a glass cyclone and cooled to room temperature. For spray agglomeration at pilot scale, the slurry is fed to the water‐cooled, two‐phase atomizer (Düsen Schlick, type 970, 0.5 mm i.d. capillary) of a custom‐made spray dryer by a syringe pump (Teledyne Isco, 1000D) at 40 mL min^−1^. It is dispersed with 30 L h^−1^ of air with a 1.7 bar pressure across the atomizer tip while a coflow of ≈0.6 Nm^3^ min^−1^ ambient air heated electrically to 200 °C (Leister heater, 6.7 kW) is established. Product particles are collected with a cyclone connected to a radial outlet at the conical bottom of the 1.8 m high, 0.5 m i.d. stainless steel spray drying chamber. Fine particles passing through the cyclone are removed by a bag filter before the off‐gas is vented with the help of a frequency‐controlled Roots blower.

The cellulose binder, PS beads, and carbon fibers are removed via calcination for one hour at 750 °C under air. Microspheres without templates (0% cellulose) were also calcined under the same conditions.

*Electrochemistry*: Electrode slurries with weight ratios of LTO to carbon black to polyvinyldifluoride binder (Solvay, Kynar 900) of 90:5:5 in *N*‐methyl pyrrolidone solvent are mixed for 6 min using a high‐shear disperser. The slurries are cast by a doctor blade with 100 μm wet thickness on a 12 μm copper foil. The films are then dried for 12 h at 80 °C under vacuum. Electrodes of 1.3 cm diameter are punched out and assembled under argon atmosphere in a coin cell type half‐cell against lithium with LP30 (1 m LiPF6 in 1:1 ethylene carbonate (EC): dimethyl carbonate (DMC)) electrolyte. The cells are cycled galvanostatically at different rates and at 1C for 100 cycles using a potentiostat (Astrol, Switzerland).

*Simulation*: In COMSOL, we use the LIB interface to simulate: 1) the electronic conduction in the electrode, 2) the ionic charge transport in the electrodes, the electrolyte, and the separator, and 3) the kinetics at the surface between the particle and the electrolyte.^36^ Since we are simulating an electrode consisting of a single particle, we do not input porosity into the LIB interface, which would give a homogenized description of porosity throughout the electrode volume. Rather we use the structure of the particle, which implicitly gives a porosity, and the Transport of Diluted Species Interface to solve Fick's Law for diffusion of lithium ions inside the microsphere. Equations and parameters are provided in the Supporting Information.

## Supporting information

As a service to our authors and readers, this journal provides supporting information supplied by the authors. Such materials are peer reviewed and may be re‐organized for online delivery, but are not copy‐edited or typeset. Technical support issues arising from supporting information (other than missing files) should be addressed to the authors.

Supplementary

## References

[1] J. M. Tarascon , M. Armand , Nature 2001, 414, 6861. 10.1038/35104644 11713543

[2] T. Ohzuku , R. J. Brodd , J. Power Sources 2007, 174, 449.

[3] M. Li , X. Hou , Y. Sha , J. Wang , S. Hu , X. Liu , Z. Shao , J. Power Sources 2014, 248, 721.

[4] Y. Yao , M. T. McDowell , I. Ryu , H. Wu , N. Liu , L. Hu , D. Nix , Y. Cui , Nanoletters 2011, 11, 2949. 10.1021/nl201470j 21668030

[5] M. Ebner , F. Marone , M. Stampanoni , V. Wood , Science 2013, 342, 716. 24136360 10.1126/science.1241882

[6] J. Haetge , P. Hartmann , K. Brezesinski , J. Janek , T. B. Brezesinski , Chem. Mater. 2011, 23, 4384.

[7] G. Hasegawa , K. Kanamori , T. Kiyomura , H. Kurata , K. Nakanishi , T. Abe , Adv. Energy Mater. 2014, 1400730.

[8] C. Jiang , M. Ichihara , I. Honma , H. Zhou , Electrochim. Acta 2007, 52, 6470.

[9] B. Ziebarth , M. Klinsmann , T. Eckl , C. Elsässer , Phys. Rev. B 2014, 89, 174301.

[10] E. Kang , Y. S. Jung , G. H. Kim , J. Chun , U. Wiesner , A. C. Dillon , J. K. Kim , J. Lee , Adv. Funct. Mater. 2011, 21, 4349.

[11] H.‐G. Jung , J. Kim , B. Scrosati , Y.‐K. Sun , J. Power Sources 2011, 196, 7763.

[12] H.‐G. Jung , S.‐T. Myung , C. S. Yoon , S.‐B. Son , K. H. Oh , K. Amine , B. Scrosati , Y.‐K. Sun , Energy Environ. Sci. 2011, 4, 1345.

[13] H.‐G. Jung , M. W. Jang , J. Hassoun , Y.‐K. Sun , B. Scrosati , Nat. Commun. 2011, 2, 516. 22044995 10.1038/ncomms1527

[14] M. Armand , J. M. Tarascon , Nature 2008, 451, 7179. 10.1038/451652a 18256660

[15] P. G. Bruce , B. Scrosati , J. M. Tarascon , Angew. Chem. 2008, 47, 16. 10.1002/anie.200702505 18338357

[16] Y. Hu , Y. Zhou , J. Wang , Z. Shao , Mater. Chem. Phys. 2011, 129, 296.

[17] W. Luther , Industrial Application of Nanomaterials—Chances and Risks, VDI Technologiezentrum GmbH, Düsseldorf, Germany 2004.

[18] M. Ebner , D.‐W. Chung , R. E. García , V. Wood , Adv. Energy Mater. 2013, 4, 1301278.

[19] Y.‐S. Lin , J.‐G. Duh , J. Power Sources 2011, 196, 10698.

[20] H. Yan , Z. Zhu , D. Zhang , W. Li , Qilu , J. Power Sources 2012, 219, 45.

[21] A. Carne‐Sanchez , I. Imaz , M. Cano‐Sarabia , D. A. Maspoch , Nat. Chem. 2013, 5, 203. 23422562 10.1038/nchem.1569

[22] K. Okuyama , I. W. Lenggoro , Chem. Eng. Sci. 2003, 58, 537.

[23] G. Majano , A. Restuccia , M. Santiago , J. Perez‐Ramirez , CrystEngComm 2012, 18, 5985.

[24] F. Iskandar , I. W. Lenggoro , B. Xia , K. Okuyama , J. Nanopart. Res. 2001, 3, 263.

[25] S.‐J. Cho , F. C. Bonhomme (Johnson Controls Technology LIc), *US20130327993 A1*, 2013.

[26] K. Masters , Spray Drying Handbook, 5th ed., Longman Scientific & Technical, Harlow, UK 1994.

[27] W. Liu , P. Gao , Y. Mi , J. Chen , H. Zhou , X. Zhang , J. Mater. Chem. A 2013, 1, 2411.

[28] G.‐N. Zhu , H.‐J. Liu , J.‐H. Zhuang , C.‐X. Wang , Y.‐G. Wang , Y. Y. Xia , Energy Environ. Sci. 2011, 4, 4016.

[29] C. Zhu , G. Saito , T. Akiyama , J. Mat. Chem. A 2013, 1, 7077.

[30] F. Iskandar , Mikrajuddin , K. Okuyama , Nano Lett. 2001, 1, 231.

[31] F. Iskandar , Mikrajuddin , K. Okuyama , Nano Lett. 2002, 2, 389.

[32] J. Kim , O. Wilhelm , S. E. Pratsinis , Adv. Eng. Mater. 2002, 4, 494.

[33] J. Kim , O. Wilhelm , S. E. Pratsinis , J. Am. Ceram. Soc. 2001, 12, 2802.

[34] T. Ohzuku , A. Ueda , N. Yamamota , J. Electrochem. Soc. 1995, 142, 1431.

[35] S. B. Pope , Cornell University Report FDA 08‐01 2008.

[36] M. Doyle , J. Newman , A. S. Gozdz , C. N. Schmutz , J. M. Tarascon , J. Electrochem. Soc. 1996, 143, 1890.

