# Supplementary material for: Design and Fabrication of Microspheres with Hierarchical Internal Structure for Tuning Battery Performance
Source: Adv Sci (Weinh). 2015 Apr 21;2(6):1500078. doi: 10.1002/advs.201500078 (PMC5016769; doi:10.1002/advs.201500078)
Supplement: Supplementary file 1 — Supplementary [file ADVS-2-0g-s001.pdf]

Copyright WILEY-VCH Verlag GmbH & Co. KGaA, 69469 Weinheim, Germany, 2015.

ADVANCED SCIENCE
Open Access

# Supporting Information

for *Adv. Sci.*, DOI: 10.1002/advs.201500078

# Design and Fabrication of Microspheres with Hierarchical Internal Structure for Tuning Battery Performance

Lea V. Nowack, Teutë Bunjaku, Karsten Wegner, Sotiris E. Pratsinis, Mathieu Luisier, and Vanessa Wood*

Copyright WILEY-VCH Verlag GmbH & Co. KGaA, 69469 Weinheim, Germany, 2013.

# Supporting Information

**Design and Fabrication of Microspheres with Hierarchical Internal Structure for Tuning Battery Performance**

*Lea.V. Nowack, Teute Bunjaku, Karsten Wegner, Sotiris.E. Pratsinis, Matthieu Luisier, Vanessa C. Wood**

<u>Comparison of Particles Before and After Calcination</u>

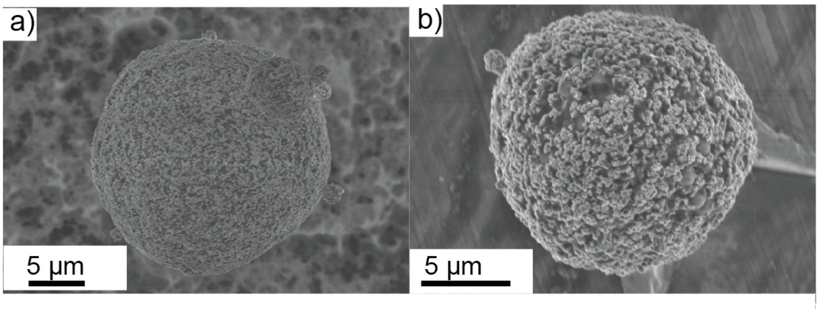


Figure S1 SEM images of particles with (a) 5% cellulose and (b) PS beads prior to annealing.

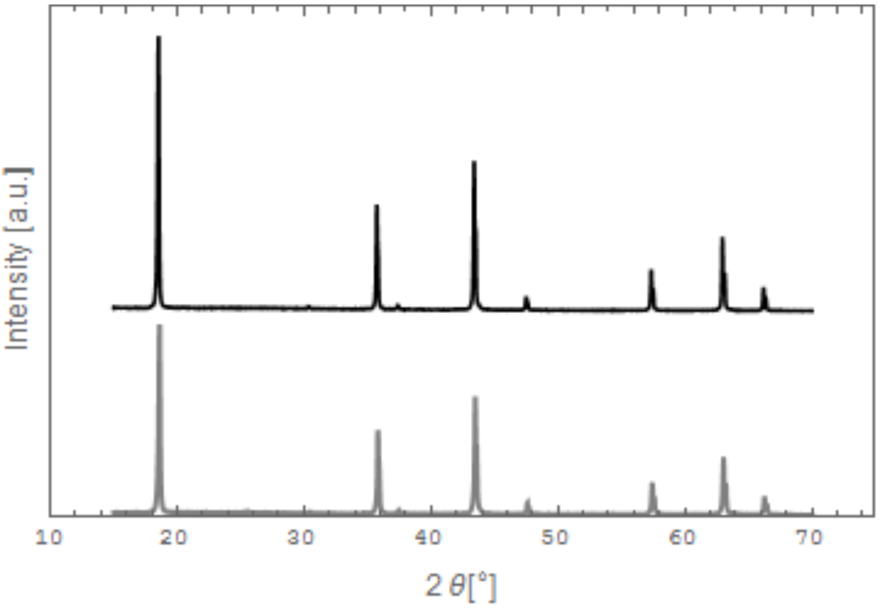


**Figure S2** XRD pattern of LTO nanopowder as received (grey) and LTO sintered microparticles after one hour calcination in air at 750°C (black). Analysis of the diffraction pattern by refinement indicates an average crystal size of 121 nm before and 183 nm after calcination.

We work with commercial nanoparticles and therefore expect excellent cycle stability; however, we test long term cycle stability of our nanoporous particles.

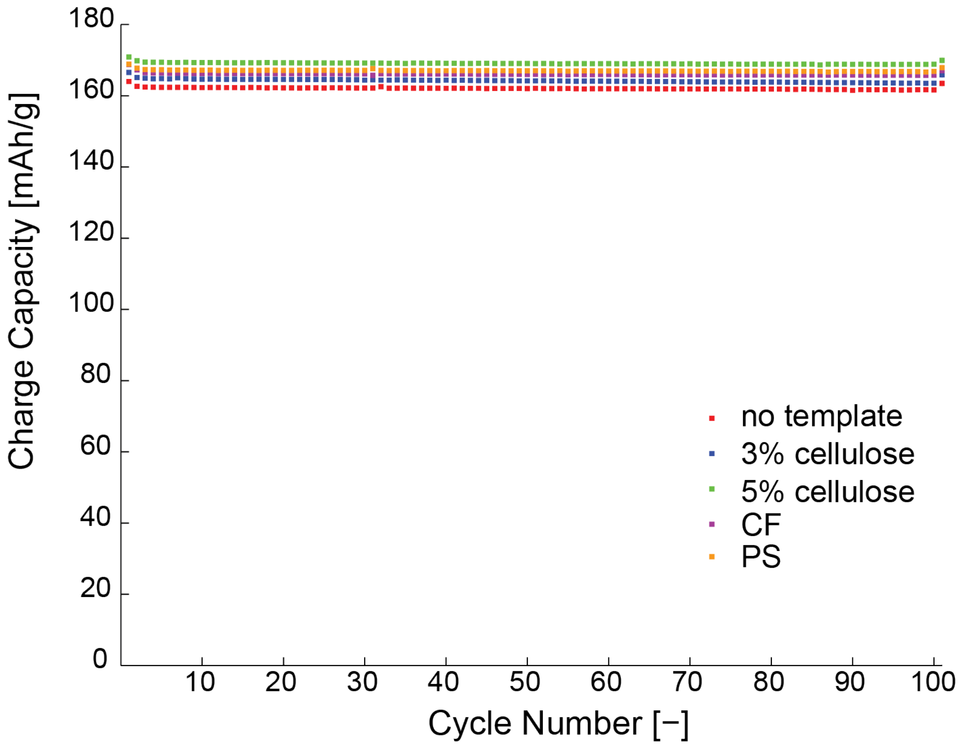


**Figure S3** *Long term cycling stability of the obtained structures at 1C. The first and the last cycle are carried out at C/10.*

Electrochemical Simulations

The simulations for the 2D and 3D single particles are performed using the Batteries and Fuel Cells Module of COMSOL Multiphysics. Two interfaces of the Electrochemistry Module are used: the Lithium-Ion Battery Interface and the Transport of Diluted Species Interface. The simulation environment is shown in **Figure S4** and the parameters are tabulated in **Table S1**.

*LTO particle*

Electronic current conduction in the particle is given by Ohm's law:

$$\boldsymbol{i_s} = -\sigma_s \nabla \phi_s, \tag{1}$$

where $\sigma_s$ is the electronic conduction coefficient of the electrode material and $\phi_s$ the particle potential. During lithiation, spinel $Li_4Ti_5O_{12}$ is converted to rocksalt phase $Li_7Ti_5O_{12}$ and the

electrical conductivity is dramatically enhanced to $10^{-2}$ $Scm^{-1}$.[37,38] The diffusion of the lithium ions inside the microsphere is found by solving Fick's Law:

$$\frac{\partial c_s}{\partial t} = \nabla(D_s \nabla c_s) + R_s, \tag{2}$$

$$\boldsymbol{N_s} = -D_s \nabla c_s, \tag{3}$$

where $D_s$ is the diffusion coefficient of the lithium ions in the LTO, $c_s$ the lithium ion concentration in the LTO, and $\boldsymbol{N_s}$ the flux.

*Electrolyte*

The initial lithium ion concentration in the electrolyte is 1 mol $l^{-1}$ to agree with the experimental $LiPF_6$ salt concentration. The differential equation governing mass balance for the salt concentration in the electrolyte $c_l$ is given by

$$\frac{\partial c_l}{\partial t} = \nabla(D_l \nabla c_l) - \frac{\nabla \boldsymbol{i_l} t_+}{F} + R_l, \tag{4}$$

$$t_+ + t_- = 1, \tag{5}$$

where $R_l$ is the total lithium ion source term, which is defined by the applied current density, $D_l$ is the diffusion coefficient for the electrolyte, and *F* is the Faraday constant. The transport number $t_+$ describes the fraction of charge carried in an electrolyte by the positively charged lithium ions. The current for lithium in the electrolyte is given by Ohm's law with a concentration variation:

$$\boldsymbol{i_l} = -\sigma_l \nabla \phi_l + \frac{2\sigma_l RT}{F}\left(1 + \frac{\nabla \ln(f)}{\nabla \ln(c_l)}\right)(1 + t_+)\nabla \ln(c_l), \tag{6}$$

where $\phi_l$ is the electrolyte potential, and $f$ is the activity coefficient for the salt, which is a factor to account for deviations from the ideal behavior in a mixture of chemical substances.

*Electrolyte-Particle Interface*

The Butler-Volmer equation, which relates gives the current density in the electrode for an applied potential $E_0$ and, is solved at the particle-electrolyte interface:

$$j = j_0\left[exp\left(\frac{\alpha_a F(\eta-E_0)}{RT}\right) - exp\left(-\frac{\alpha_c F(\eta-E_0)}{RT}\right)\right], \tag{7}$$

$$j_0 = Fk_c^{\alpha_a}k_a^{\alpha_c}\left(c_{s,max} - c_s\right)^{\alpha_a}c_s^{\alpha_c}c_l^{\alpha_a}. \tag{8}$$

The exchange current density is represented by $j_0$, where $k_c$ and $k_a$ are the rate coefficients for the cathodic respectively the anodic reaction, $\alpha_c$ and $\alpha_a$ represent the transfer coefficients for the cathodic and anodic reaction. The value $c_s$ is the lithium ion concentration inside the electrode particle, and the maximum amount of lithium ions inside the electrode is defined by $c_{s,max}$. The overpotential $\eta$ is the difference between the electrode and electrolyte potential $\eta = \phi_s - \phi_l$.

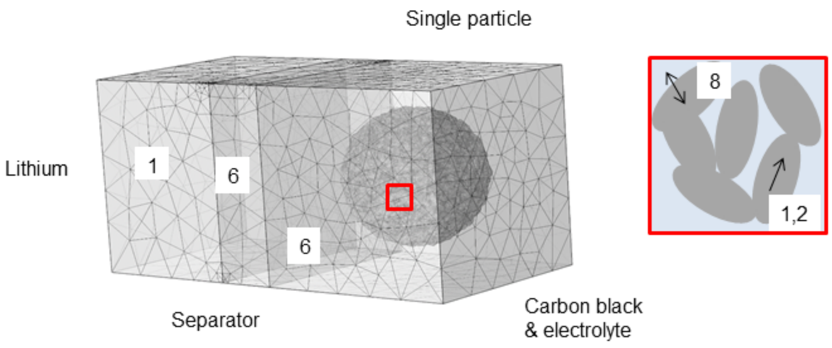


**Figure S4** The simulation environment created in COMSOL Multiphysics consists of a single particle made of ellipsoids. Numbers in the figure correspond to the numbers of equations in the text that are used to describe the interfaces and environments.

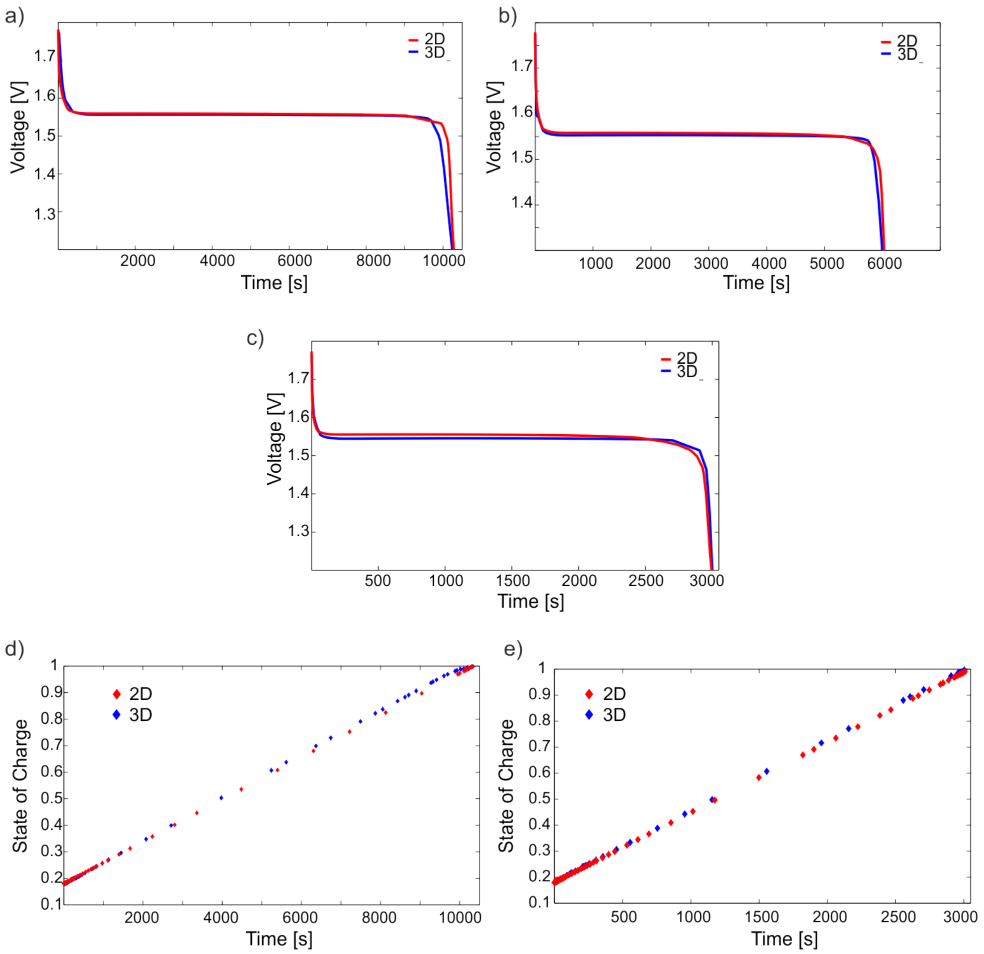


**Figure S5** Comparison of discharge curves obtained by simulations on a 2D (red) and 3D (blue) structure each with 41% porosity at a a) 1 C b) 2.5C and c) 5C rate. Comparison of state of charge (SOC) at different time steps during d) 1C and e) 5C rate of the same structure in 2D and 3D.

**Table S1.** Values used in simulation

| Parameter | Value |
|---|---|
| Diffusion coefficient particle $D_s$ | 5x10$^{-17}$ m$^2$s$^{-1}$(delithiated) and 1.5x10$^{-14}$ m$^2$s$^{-1}$ (lithiated) |
| Conductivity particle $\sigma_s$ | 10$^{-9}$ – 1 Sm$^{-1}$ |
| Anodic/cathodic transfer coefficient *t* | 0.5 |
| Rate constant $k$ | 2x 10$^{-11}$ ms$^{-1}$ |
| Max. lithium concentration $c_{s,max}$ | 23720 molm$^{-3}$ |
| Initial lithium concentration particle | 4270 molm$^{-3}$ |
| Diffusion coefficient electrolyte $\sigma_l$ | 2.5x 10$^{-10}$ m$^2$s$^{-1}$ |
| Conductivity carbon/electrolyte mix $\sigma_{mix}$ | 5.62 Sm$^{-1}$ |

[37] N. Takami, K. Hoshina, H. Inagaki, *J. Electrochem. Soc.* **2011**, 6, A725.
[38] D. Young, A. Ransil, R. Armin, Z. Li, Y.-M. Chiang, *Adv. Energy Mater.* **2013**, 3, 1125.
